# Draft Genome Sequences of Four Rhodobacter sphaeroides Strains Isolated from a Marine Ecosystem

**DOI:** 10.1128/MRA.01648-18

**Published:** 2019-01-17

**Authors:** Michael S. Guzman, Arpita Bose

**Affiliations:** a Department of Biology, Washington University in St. Louis, St. Louis, Missouri, USA; Louisiana State University

## Abstract

Rhodobacter sphaeroides is an alphaproteobacterium found in freshwater and marine ecosystems. To better understand the metabolic diversity within this species, we isolated and sequenced four R. sphaeroides isolates obtained from Trunk River in Woods Hole, Massachusetts. Here, we report the draft genome sequences of R. sphaeroides AB24, AB25, AB27, and AB29.

## Abstract (precis)

Rhodobacter sphaeroides is an alphaproteobacterium found in freshwater and marine ecosystems. To better understand the metabolic diversity within this species, we isolated and sequenced four R. sphaeroides isolates obtained from Trunk River in Woods Hole, Massachusetts.

## ANNOUNCEMENT

Rhodobacter sphaeroides is a model purple nonsulfur bacterium for studying microbial metabolism and bioenergetics (1, 2). It is exceptionally metabolically versatile, being capable of photoheterotrophy, photoautotrophy, chemoheterotrophy, chemoautotrophy, and fermentation. R. sphaeroides has also been investigated for its biotechnological (3, 4) and bioremediation (5 – 8) potential. To date, only 13 R. sphaeroides genome sequences have been deposited in the GenBank database. To examine the genetic diversity of natural isolates within this species, we isolated and sequenced the genomes of four R. sphaeroides strains from Trunk River in Woods Hole, Massachusetts. We determined that these isolates are 99% similar to R. sphaeroides KD131 (9) based on 16S rRNA gene sequence analysis.

Seawater was sampled from Trunk River, and 500 μl was used as an inoculum into Pfennig bottles containing anoxic artificial seawater medium (10) supplemented with 20 mM acetate. Enrichments were cultivated with ∼850-nm light at 30°C and passaged six times in anoxic artificial seawater medium, followed by streaking oxically 6 times on Bacto agar with Difco marine broth 2216 (BD Diagnostic Systems, Sparks, MD, USA). Genomic DNA was isolated with the DNeasy blood and tissue kit according to the manufacturer’s recommendations (Qiagen, Dusseldorf, Germany) from single colonies cultivated in marine broth to mid-log phase. Paired-end 250-bp Illumina sequencing libraries were prepared using the Nextera sample prep kit (San Diego, CA) and sequenced on a MiSeq instrument using v2 chemistry (Illumina, Inc.) to 300× (AB24), 38× (AB25), 36× (AB27), or 33× (AB29) coverage. Reads were trimmed with Trimmomatic version 0.38 with the program’s default parameters for paired-end reads (11). The trimmed reads were *de novo* assembled with SPAdes version 3.13.0 using the program’s default parameters (12). Contigs were extended using the reference-guided scaffolder MeDuSa version 1.6 with the complete genome of R. sphaeroides KD131, using the program’s default parameters (13). Alignment of the scaffolded genomes of AB24, AB25, AB27, and AB29 was performed with LASTZ version 1.02.00 to examine plasmid and chromosomal synteny between the isolates and reference genomes (14). Sequences were submitted for annotation to the NCBI Prokaryotic Genome Annotation Pipeline (PGAP) (15). The identity of each strain was determined by analyzing the full-length 16S rRNA gene sequence predicted by PGAP in the genome assemblies. NCBI BLASTN analysis was performed on these sequences to determine the identity using the program’s default parameters (16). Phylogenetic analysis was performed with the BLASTN alignments using the BLAST Tree View widget, with the program’s default parameters (http://blast.ncbi.nlm.nih.gov/).

The genomes of AB24, AB25, AB27, and AB29 have a total assembly length of ∼3.2 Mb (Table 1) and a GC content of 69.1%. Genome scaffolding produced four sequences for each strain (Table 1) that mapped to chromosomes I (3.2 Mb) and II (1 Mb), as well as plasmids A (0.1 Mb) and B (0.2 Mb), of R. sphaeroides KD131 and R. sphaeroides 2.4.1, as determined by synteny analysis (17). Neither contigs nor sequencing reads mapped to plasmid C, D, or E of R. sphaeroides 2.4.1, similar to R. sphaeroides KD131. NCBI PGAP predicted 4,280 (AB24), 4,271 (AB25), 4,086 (AB27), and 4,169 (AB29) open reading frames. The isolates contain multiple copies of genes involved in DNA replication, amino acid metabolism, motility and chemotaxis, photosynthetic light harvesting, and central carbon metabolism, as has been characterized in R. sphaeroides 2.4.1 (1, 17). The isolates encode proteins involved in lithotrophic metabolism (Ni-Fe uptake hydrogenase and CO dehydrogenase), nitrogen fixation (Fe-Mo nitrogenase), and denitrification (nitrous oxide and nitric oxide reductase) (5, 18 – 20). These genomes provide opportunities for future studies into the metabolic potential of R. sphaeroides in marine ecosystems.

**TABLE 1:** Genome statistics and accession numbers

Strain	No. of reads	Assembly size (Mb)	No. of scaffolds	SRA accession no.	GenBank accession no.
AB24	2,899,454	3.26	4	SRR8107695	CP033434–CP033437
AB25	676,996	3.23	4	SRR8107697	CP033442–CP033445
AB27	637,638	3.23	4	SRR8107696	CP033446–CP033449
AB29	569,874	3.24	4	SRR8107698	CP033450–CP033453

### Data availability.

These whole-genome shotgun (WGS) projects and the raw sequencing reads have been deposited in GenBank and the NCBI Sequence Read Archive (SRA), respectively, under the accession numbers listed in Table 1.
